# Sustainable application of novel herbs on cotton fabrics as biomordants and colourants

**DOI:** 10.1007/s11356-022-18818-7

**Published:** 2022-02-19

**Authors:** Alka Madhukar Thakker, Danmei Sun

**Affiliations:** School of Textile and Design, Heriot-Watt University, TD1 3HF Scottish Borders, UK

**Keywords:** Plant-based colours, Herbal fabrics, UV protective fabrics, Plant-based copper, Plant-based ferrous

## Abstract

The textile industry is ambitiously parading towards circularity and curtailing its dependency on fossil fuels hence the instigated research that adheres to Sustainable Developmental Goals (SDGs) and benefits the environment and human health alike. The current research experimented with innovative sources of plant-based biomaterials namely dandelion leaves, bilberry leaves, nettle leaves, and violet herb for application on cotton fabric as biomordants and colourants. The AAS analysis of mild 6% herbal solution revealed ferrous content of 6.78 mg/l in violet herb and 5.03 mg/l of the copper content in dandelion leaves applicable on cotton fabrics as biomordants thereby preventing the depletion of copper and ferrous elements mineral ores. A fair lightfastness rating and good to very good wash and rub fastness test results were obtained individually. The maximum K/S value of 13.95 was gained on cotton fabrics treated with a mild herbal infusion of bilberry leaves and coloured with violet herbs. The ATR-FTIR peak analysis noted strong = C-H bend of alkenes, strong C = O stretch of α and β unsaturated esters, and strong C-O stretch of carboxylic acids functional groups in raw herbs and its treated cotton fabrics. The dissolved oxygen (DO), oxidation–reduction potential (ORP), and potential of Hydrogen (pH) values were found in the acceptable range for all the spent solutions left after colouration of cotton fabrics with violet herbs hence sustainable. The data set obtained was statistically validated with ANOVA one-way test. Life cycle analysis and clinical investigation on potential therapeutic benefits of herbal fabrics to the wearer are suggested for future research and development.

## Introduction

Data from several studies suggest that it is imperative to acquire sustainability in textile wet processing units. Therefore, alternative herbal materials would enable detoxifying textiles. The plant-based sources of mordants and colourants are primarily fallen stems, barks, and leaves. They are biodegradable at the molecular stage and renewable. The herbal raw materials do not generate emissions or effluents as synthetic counters hence safe (Sharma et al. 2018; United States Environmental Protection Agency 2020). Furthermore, the research studies on colours from herbs for textiles indicate no potential hazard or toxicity to humans and animals’ well-being (Vankar and Shukla 2019). The spent solution after processing organic fabrics with herbal colours is a zero-carbon footprint and could be upscaled readily. The surplus herbal solution could be safely released into the land or water areas with no harm done to the surrounding flora, fauna, or human health (Neddo 2015). Still, to evident the theory, prospective research is being proposed. Subsequently, the metal-based mordants that deplete the mineral ores and are poisonous to the marine flora (Horne and Goldman 1994) and fauna are required to be replaced by plant-based renewable mordants for sustainable colouration with renewable plant-based colours. Thus, the envisaged research investigates innovative plant-based sources of ferrous and copper mordants from surplus nettle leaves, dandelion leaves, and bilberry leaves for application on cotton fabric. The biomordanted fabrics were further ecologically treated with natural colourant from violet herb leaves. The samples were quantitatively analyzed.

Water pollution from fossil-based textile wet processing units especially the colouration sector releases massive amounts of toxic chemicals in the surrounding environment enormously damaging adjacent flora and fauna including human beings (Christie 2015; Greenpeace International 2018). The link to a video, https://www.scmp.com/video/asia/3103059/not-single-family-healthy-indian-village-sickened-contaminated-water, would deeply affect and awaken the world civilization towards the well-being of the earth, environment, and humans on an urgent note (South China Morning Post Publishers Ltd. 2021). The emissions and effluents from textiles industries are the second largest in the world next to big oil. An Ayurvedic colouration is an ecological approach towards textile colouration that would propel sustainability in a game-changing way. Hence, the hypothesized research is set on this. Subsequently, propelled by the global sustainability agenda, an upsurge in research studies concentrated on the application of biomordants on the natural textile substrate was noted as cited in Table 1.

**Table 1:** Critical analysis of research studies implementing natural mordants on natural textile substrates (Habib et al. 2021; Souissi et al. 2018; Shahid et al. 2019; Mohd et al. 2019; Geelani et al. 2016; Xiaocui and Chen 2019; Iqbal and Patel 2018)

Bio mordant	Substrate	Colour	Technique	Result	Critical analysis
Arjun bark, zeera, lili elchi, harmal, neem	Cotton	Reddish-yellow	Ultrasonic bath, CIELa*b*, (Habib et al. 2021)	-	Acidic Methanolic extraction, hence not ecological, Fastness properties were overlooked
Date palm pits, gall walnut, green almond shells, chlorophyll	Cotton fibres	Brown, Beige	Parametric experiment, CIE L a* b* BOD, COD, ANOVA	A very good wash, rub, perspiration, and lightfastness (Souissi et al. 2018)	Phytochemical analysis on FTIR and functionality test on UV–vis spectrophotometer were not provided
Pomegranate peels, catechu, gallnut, Palash	Wool yarns	Brown, green, burgundy, yellow	CIE L a* b*	A good wash and rub fastness (Shahid et al. 2019)	Too high M:L ratio of 1:40, high temperature 93 °C Did not provide BOD and COD results
Ratanjot, gallnut, pomegranate peels, babool	Wool yarns	Yellow	CIE L a* b* SEM	A good wash, rub, and lightfastness (Mohd et al. 2019)	Too high temperature of 90 °C, FTIR, UV–vis’s analysis was not provided
Fruit cups, white willow, cottonwood	Wool cotton silk pashmina	Brown, off white	UV–vis CIE L a* b*	good wash and lightfastness (Geelani et al. 2016)	Too high M:L ratio of 1:60, BOD and COD were not provided
Tea, turmeric, tannic acid	Silk	Yellowish-brown	Ultrasonic bath CIE L a* b*	good wash and lightfastness (Xiaocui and Chen 2019)	95% ethanol extraction, too high M:L ratio of 1:50, high temperature of 80 °C
Turmeric, eucalyptus, myrobalan, pomegranate rind	Cotton	Dark yellow Khaki	Fastness tests	Good wash fastness (Iqbal and Patel 2018)	Details on methods, CIELa*b* and further analysis were not provided

Additionally, the study conducted by Roman found that *Posidonia oceanica* fibres pre-treated with medium chitosan (natural biomordant) showed the highest intensity of colour on the reflection spectrophotometer (Roman et al. n.d.). The research overlooks K/S values, fastness properties, and functional properties of chitosan. A red natural dye utilized for the study is not specified at all. Researchers have not treated biomordants in much detail. Overall though, there seems to be some evidence that biomordants play a vital role in generating sustainable ecological textiles. Interestingly, nettle leaves extraction was utilized by Eser and Onal for the colouration of cotton and wool fabrics successfully. The fabrics were mordanted with artificial animal urine, copper sulfate, and ferrous sulfate (Eser and Onal 2015). In the current world climate crisis, the implementation of fossilized mordants depletes the mineral reserves and hence not ecological. Also, Hosseinnezhad et al. recently performed a study concluding tannin-rich oak bark as an efficient natural mordant for wool yarns for colouration with madder and weld. A very good wash fastness rating was obtained (Hosseinnezhad et al. 2021).

The concept of sustainability is at its forefront worldwide. Common sense would be to realize that sustainability is here to stay, it is not a fashion fad. It conscientiously comprises environmental, social, and governance (ESG) and corporate social responsibility (CSR) (Picton and Nicola 2021). Plant-based innovative sources of materials for colouration and colour fixation aka ayurvedic colours for fabrics would propel sustainability in the field of the textile industry (Ayurvastra 2020). Concerning, the ultraviolet protection functionality of the herbal fabrics (UVP), the previous study by Sarkar evaluated UPF of cotton fabrics coloured with madder, indigo, and cochineal, and resultant fabrics were concluded to be excellent in UVP ability. Likewise, it was established that darker hues with a high concentration of natural colour on the fabric are more UV protective (Sarkar 2004). In the same vein fabric, colours with L* values below 38 are more UV protective (Wilson et al. 2008). Concurrently, the colouration of cotton, wool, silk, and nylon fabrics with madder, red onion peels, and chamomile yield excellent UVF values except for nylon fabric coloured with madder. Hence, natural-coloured fabrics were recommended for preventing skin cancer (Gawish et al. 2016). However, it requires further clinical investigations to support the statement. Therefore, the envisaged research is determined to promulgate the innovative sources of herbs for application on cotton fabrics with UVP functionality.

In the same vein, Thakker and Sun examined native flora, namely mugwort herb, rue herb, and black cherry stem as biomordant for application on cotton fabrics, and sequentially coloured with hops flowers. The atomic absorption spectrometry (AAS) analysis of 6% stock herbal infusions identified the highest levels of copper and ferrous content in hops flower, and black cherry stems of 6.5 mg/l and 11 mg/l, respectively, as a prospective ecological and innovative source of mordants and colours. It was noted that the UV transmittance has markedly dropped for the cotton fabrics pre-treated with biomordants and coloured with hops flowers as compared to the original cotton fabric. Therefore, the treated fabrics are suitable for summer wear. The cotton fabrics pre-treated with black cherry stems and coloured with hops flowers exhibited the minimum transmittance percentage in the UVB region at 12.98%. Similar is the envisaged research (Thakker and Sun 2021a, b). The method of extraction, mordanting, and colouration was low on water and energy demand. The method devised impels original colour stability and phytochemical stability that would be otherwise destroyed due to high temperature and high material and liquor (M:L) ratio (Thakker and Sun 2021a, b).

There is no scientific documentation of the application of herbs, namely dandelion leaves, bilberry leaves, nettle leaves, and violet herb on cotton fabric. However, there exists ancient grey literature that mentions the colouration of cotton and wool fabrics with bilberry leaves, nettle leaves, and violet herb to obtain green colour with synthetic mordants (Just Ingredients 2021), consequently the initiated research. The research expounded adhere to cradle-to-cradle theory, citing “sustainability and circularity start with material identity”. Eco-effective textiles from natural raw materials namely natural fibres, natural colours, and natural additives should be introduced to a biological cycle as nutrients after use. “Complete transparency of all ingredients” would be mandatory (EPEA GmbH 2020).

## Materials and methods

### Materials

Cotton fabric was sourced from Whaley’s Bradford Limited, UK, specified in Table 2. Raw herbal materials in this research, namely dandelion leaves, nettle leaves, bilberry leaves, and violet herb, were obtained from Just Ingredients Limited, UK; refer to Table 3 for plant profile and their potential functionality on textiles substrate.

**Table 2:** Cotton fabric material details

Fabric	Weave structure	Fibre type	Yarn count, tex		Density		Fabric weight, g/m^2^
			warp	weft	Warp, ends/5 cm	Weft, picks/5 cm	
Calico (CF/OC/C)	Plain weave	100% cotton	30	30	60	60	140

**Table 3:** Profile of plants in research as a source of sustainable mordants

Plant name	*Botanical name*	Common name	Part of plant	Potential functionality
Oak bark (OB) Standard	*Quercus pedunculata*	Royal oak, Duir, White oak, English oak	Bark	Anti-microbial, anti-inflammatory
Dandelion leaves (DL)	*Taraxacum Officinalis*	Dandelion, Clock flower, Tell-The-Time, Blowball, Puffball, Priest’s Crown	Leaves	Anti-inflammatory
Nettle leaves (NL)	*Urtica dioica*	Common Nettle, Ortie, Urtiga, Chichicaste	Leaves	Anti-inflammatory
Bilberry leaves (BL)	*Vaccinium myrtillus*	Bilberry, Huckleberry, Hurtleberry	Leaves	Anti-inflammatory, anti-microbial
Violet herb (VH)	*Viola tricolour*	Pensée, Pensiero, Trilliw, Heartsease, Banewort	Leaves	Skin infection

### Methods

The sustainable techniques of treating the cotton fabrics with herbs were adopted for ecological outcomes as elaborated further.

#### Process of extraction

**Fig. 1:**
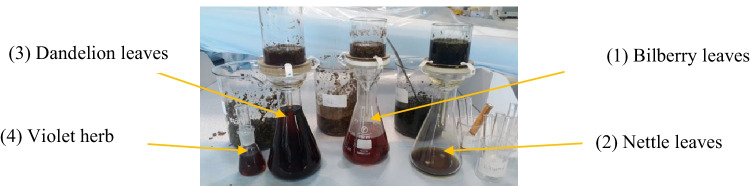
The extraction process of herbs in research

The 30 g of raw herbal leaves was infused in 500 ml of distilled water at 60℃ and steeped for 2 h for the extraction process to complete sustainably. The extract was filtered with Whatman glass microfibre filters of 70 mm. The prepared 6% of the stock solution was further utilized for cotton fabric treatment; refer to Fig. 1.

#### Method of mordanting

For 30% strength of treatment, 6% of the herbal mordant extract stock solution (owf, on the weight of fabric) of each oak, bark, dandelion leaves, bilberry leaves, and nettle leaves were utilized for soaking cotton fabric samples. The fabric samples were steeped for one hour at room temperature of 25℃ with material to liquor ratio of 1:10. The treated fabrics were shade dried ready for further processing and examination. 

#### Method of colouration

The 30% depth of shade (owf) of the herbal colour extract solution prepared from the violet herb was utilized for colouring cotton fabric samples. The mordanted fabrics were soaked in the colour extract solution for 2 h at room temperature of 25℃ with material to liquor ratio of 1:10. The coloured fabric samples were shade dried. The process is simple and clean involving no emissions and effluents. The process devised propels optimal functionality and original colour stability. 

#### Colour measurement

**Fig. 2:**
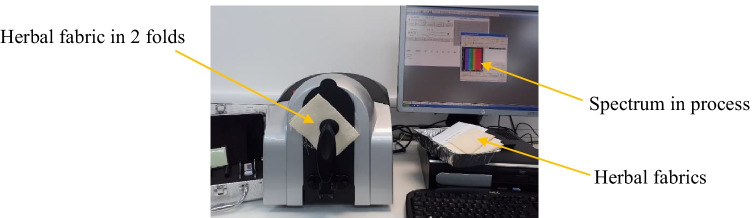
Investigation of colour values on Datacolour 600

Datacolour 600, a dual-beam spectrometer, was utilized for colour measurement of cotton fabrics pre-treated with herbs such as oak bark, dandelion leaves, bilberry leaves, and nettle leaves and subsequently coloured with the violet herb; refer to Fig. 2. The instrument has an SP2000 monochromator with dual 256 LEDs and a high-resolution holographic grid. The source of light in it is D65; it covers the spectral range from 360 to 700 nm and has a photometric range of 0 to 200% (Technical Color Solution 2012–2013).

#### Attenuated total reflectance-Fourier transform infrared spectroscopy (ATR-FTIR) analysis

**Fig. 3:**
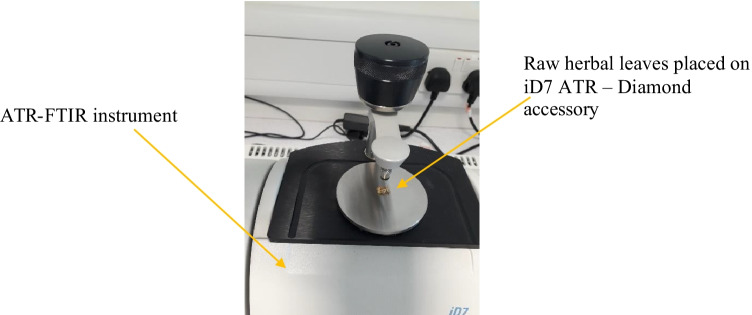
ATR-FTIR analysis of raw herbs in progress

Thermo Scientific™, Nicolet™ iS™ 5 FTIR Spectrometer was implemented for examining the constituent profile of the raw herbal leaves and the cotton fabrics treated with herbs, namely, oak bark, dandelion leaves, bilberry leaves, nettle leaves, and violet herb; refer to Fig. 3. In the FTIR principle, in infrared spectroscopy (IR), IR radiation is passed through a sample, some of the IR is absorbed by the sample, and some of it is transmitted through the sample. The resulting spectrum represents the molecular absorption and transmission, creating a molecular fingerprint of the sample. Fourier transform infrared spectrophotometers (FTIR) are mainly used to measure light absorption of so-called mid-infrared light, in the range of wavenumber between 4,000 and 400 cm^−1^ (wavelengths from 2.5 to 25 µm), to identify and quantify various materials. The ATR-FTIR technique was utilized for acknowledging the presence of predominant functional groups in each of the raw herbs, biomordanted cotton fabric samples, and coloured cotton fabric samples (Vickerman 1997) (Thermo Fisher Scientific Nicolet™ iS™ 5 FTIR Spectrometer n.d.).

#### Atomic absorption spectrometry analysis

**Fig. 4:**
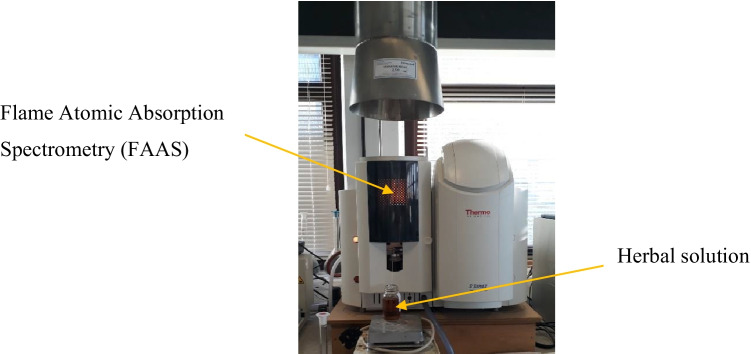
Herbal solution element test on atomic absorption spectrometry (AAS) (Thermo Fisher Scientific n.d.)

The atomic absorption spectrometry (AAS) was implemented for elemental analysis of the copper and ferrous in 6% herbal stock solution for their prospective application on cotton fabrics as ecological mordants and or colours. Thermo scientific S, series, AA spectrometer performs flame analysis, and it is a simple, single atomizer AAS with an automatic gas box; refer to Fig. 4. The AAS was calibrated before the research experiment; the working calibrating solutions were prepared from standard stock solutions which are typically 1,000 ppm in concentration. The 30% strength of herbal stock solutions of each oak bark, dandelion leaves, nettle leaves, bilberry leaves, and violet herbs were prepared in distilled water for further inspection on AAS (Thermo Fisher Scientific n.d.). The AAS is a quantitative analysis based upon the theory that free atoms in the ground state can absorb light of a specific wavelength. Absorption for each element is certain; no other elements absorb this wavelength. The wavelength range is 180 to 900 nm. The absorbance range is -0.150A to 3.000A (Thakker and Sun 2021a, b).

The flame atomic absorption spectrometry (FAAS) analysis was performed to identify the copper and ferrous content of each herb namely, dandelion leaves, bilberry leaves, nettle leaves, oak bark, and violet herb as a prospective source of biomordant that would assist in preventing the utilization of fossil-based mordants of copper and ferrous sulfates as fixatives for natural colourants, hence averting the depletion of mineral ores and toxicity to surrounding aquatic flora and fauna (Horne and Goldman 1994; Alamy Ltd. 2021). The investigation would deliver plant-based bio-degradable alternatives to synthetic non-bio-degradable mordants.

#### Ultraviolet protection functionality evaluation using UV/Vis spectrophotometer

**Fig. 5:**
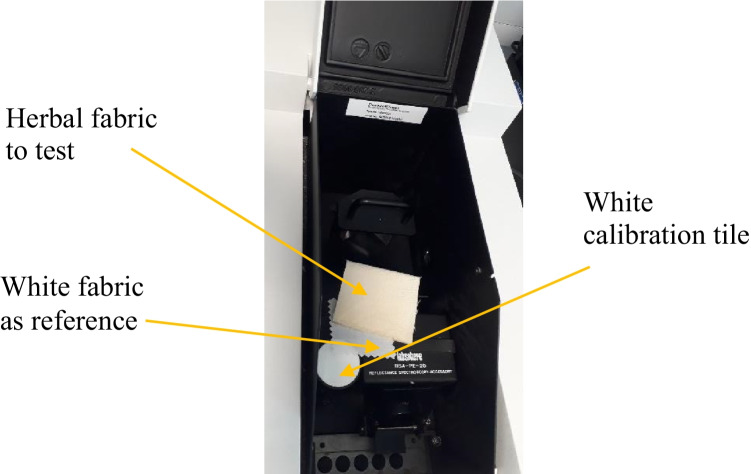
Ultraviolet, visible–spectrophotometer to examine ultraviolet protection percentage of herbal fabrics

PerkinElmer Lambda 35 UV/vis spectrophotometer was utilized to evaluate the transmittance values of the cotton fabrics treated with dandelion leaves, nettle leaves, bilberry leaves, oak bark, and violet herb, to justify their UV-protective functionality. The UV/vis spectrophotometer covers a 190–1100 nm range of wavelength with double beam operation; refer to Fig. 5. It has 0.5–4 variable bandwidth, therefore offers the best solution for measurements of solids, pastes, and powders. The treated fabrics were analyzed for the same (Perkin Elmer 2002–2011).

#### Ecological characterization of spent solutions after bio-mordanting and after colouration with herbs

The surplus solutions acquired after biomordanting and colouration of cotton fabrics with the herbs in the research were examined on selected three parameters to validate the ecological viability of the materials and methods implemented in the research.

##### Dissolved oxygen (DO)

The dissolved oxygen (DO) level of the spent solution after herbal colouration was measured with a dissolved oxygen meter Hanna, HI 9146 microprocessor. Table 4 shows the DO level requirements of marine fish in mg/l (1 mg/l = 1 ppm). The DO level could be also be expressed in ppm.

**Table 4:** Dissolved oxygen level requirement of marine fish (Horne and Goldman 1994)

Fish survival	Dissolved oxygen (mg/ l) level
All fish die	0–4
Very few fish live	4–6.5
Big fish live Small fish die	6.5–9.5
All fish live	9.5–12

##### Oxidation–reduction potential (ORP)

The surplus water after completing the mordanting and colouration process was analyzed using Hanna, HI 8424 pH/mV/℃ portable pH meter/ORP (oxidation–reduction potential) meter. The ORP is measured in millivolts (mV). The higher the ORP at the end of the process, the cleaner the water. A high ORP value indicates that there is a lot of oxygen present in the water (Horne and Goldman 1994). A positive ( +) ORP means that the solution is an oxidizing agent. A negative ( −) ORP reading means that the solution is a reducing agent (Aqua Health Products Inc 2020).

##### Potential hydrogen (pH)

pH is the quantitative measure of the acidity or basicity of an aqueous solution. It is the value of the concentration of the hydrogen ion, which ordinarily ranges between about 1 and 10^−14^ g-equivalents per litre (Encyclopaedia Britannica n.d.). Water with a pH value between 6.5 and 8.5 is considered safe for drinking, meaning the water is neither acidic nor alkaline enough to be harmful to the human body. When the pH value drops to below 6.5 or rises above 8.5, water becomes toxic, causing various health issues like eye and skin irritation, diarrhoea, nausea, and gastrointestinal upset (Center for Hazardous Substance Research (CHSR) 2006). The surplus bath after processing the cotton fabric samples with biomaterials such as oak bark, dandelion leaves, nettle leaves, and bilberry leaves and thereafter colouration with violet herb was tested for DO, ORP, and pH levels to quantify its recyclability and disposability.

#### Testing of fastness properties

Fabric samples were conditioned as per British standard (BS) EN ISO 139:2005 + A1:2011. Rub fastness test was conducted on James Heal crock meter, Model number 680 as per British standard (BS) EN ISO 105-X12:2016. A Wash fastness test was performed on SDL-ATLAS, M229 Rotawash as per BS EN ISO 105- C06:2010. Lightfastness test was conducted on Turfade, serial number 200/18/1053 as per BS EN ISO 105-B02:2014: Colourfastness to artificial light: Xenon arc fading lamp test (The British Standards Institution 2021).

## Results and discussion

Motivating and ecological outcomes as hypothesized were gained and are detailed as follows.

### Colour values as obtained on Datacolour scan 600

The results obtained on the colouration of cotton fabrics with the violet herb are tabulated in Table 5. The cotton fabric samples were biomordanted with oak bark, dandelion leaves, bilberry leaves, and nettle leaves before colouration.

**Table 5:** Colour values of mordanted & coloured cotton fabrics as obtained on Datacolour scan 600

Samples	Absorbance-scattering (K/S)	Lightness-darkness (L*)	Red–green (a*)	Yellow–blue (b*)
CF	2.80	96.26	3.19	− 14.21
VH	7.49	89.24	0.09	11.92
OB + VH	9.90	83.13	4.07	16.11
DL + VH	10.14	88.16	0.09	16.08
BL + VH	13.95	83.87	3.47	19.12
NL + VH	11.12	85.42	0.78	17.02

CF is cotton fabric, VH is violet herb, OB is oak bark, DL is dandelion leaves, BL is bilberry leaves, NL is nettle leaves

**Fig. 6:**
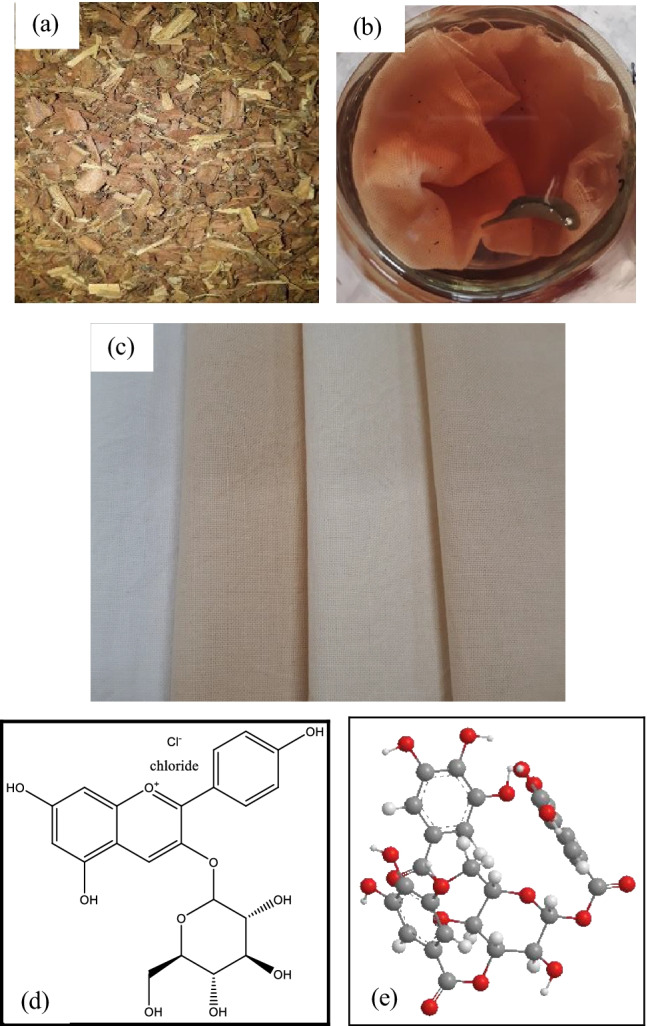
Herbal process and fabrics, **a** raw herb oak bark, **b** cotton fabric soaked in the violet herb solution, **c** cotton fabric treated with oak bark and coloured with violet herb, **d** chemical structure of anthocyanin in the violet herb (Vukics et al. 2008), and **e** the 3D model of anthocyanin (PerkinElmer Informatics 1998–2020)

**Fig. 7:**
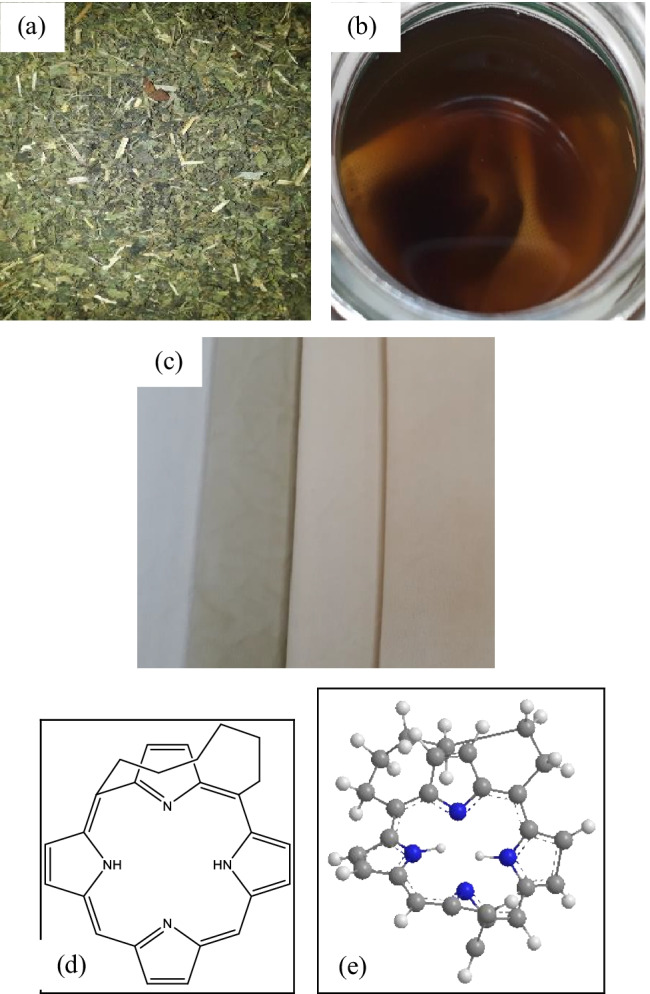
Herbal process and fabrics, **a** raw herb nettle leaves, **b** cotton fabric soaked in the nettle leaves solution, **c** cotton fabric treated with nettle leaves and coloured with Violet herb, **d** chemical structure of coproporphyrin in nettle leaves (Otles and Buket 2012), and **e** the 3D model of coproporphyrin (PerkinElmer Informatics 1998–2020)

**Fig. 8:**
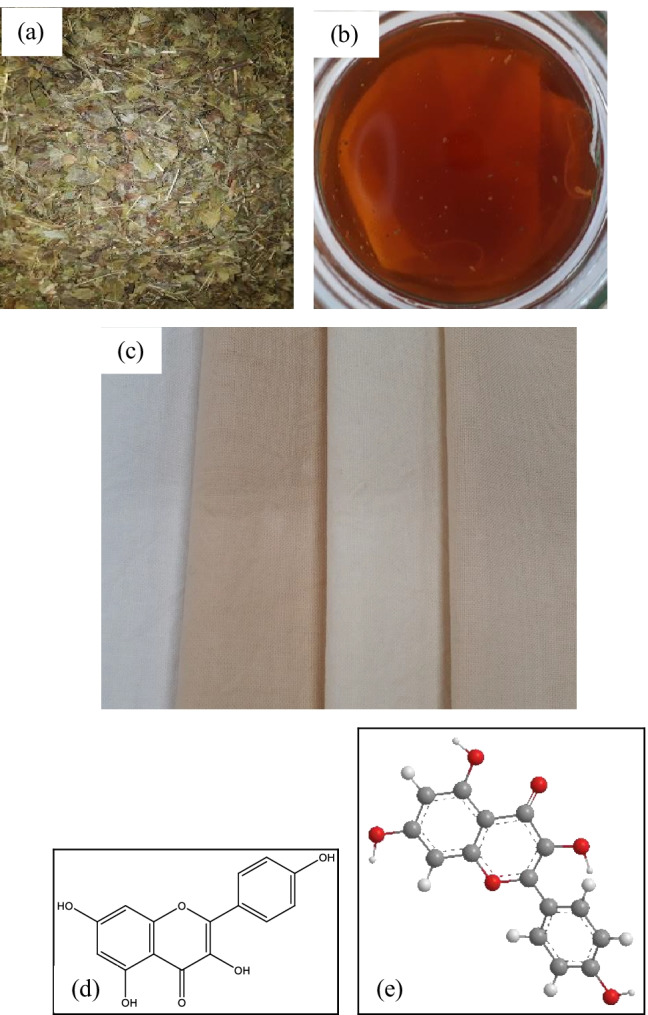
Herbal process and fabrics, **a** raw herb bilberry leaves, **b** cotton fabric soaked in the bilberry leaves solution, **c** cotton fabric treated with bilberry leaves and coloured with violet herb, **d** chemical structure of kaempferol in bilberry leaves (Stanoeva et al. 2017), and **e** the 3D model of kaempferol (PerkinElmer Informatics 1998–2020)

**Fig. 9:**
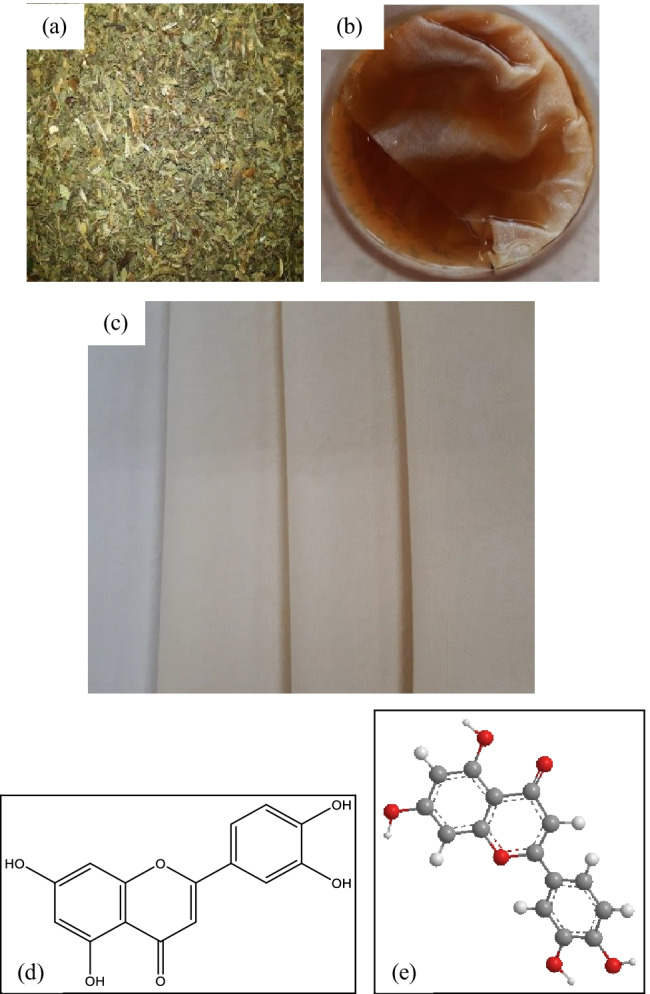
Herbal process and fabrics, **a** raw herb dandelion leaves, **b** cotton fabric soaked in the dandelion leaves solution, **c** cotton fabric treated with dandelion leaves and coloured with violet herb, **d** chemical structure of luteolin in dandelion leaves (Miłek et al. 2019), and **e** the 3D model of luteolin (PerkinElmer Informatics 1998–2020)

The maximum absorbance of 13.95 K/S was gained on cotton fabric samples pre-treated with bilberry leaves and coloured with violet herb followed by 11.12 K/S acquired on cotton fabric samples biomordanted with nettle leaves and coloured with the violet herb. The lightness values were high for all the cotton fabrics coloured with violet herb; in specific, 88.16 was obtained on dandelion pre-treated cotton fabric samples, 83.87 for bilberry leaves pre-treated fabric sample, and 85.42 for nettle leaves pre-treated fabric sample. Likewise, the sample pre-treated with bilberry leaves and coloured with violet herb gained a maximum b* value of 19.12 followed by 17.02 for fabric sample biomordanted with nettle leaves and 16.08 for fabric sample biomordanted with dandelion leaves and coloured with the violet herb. Overall, the results indicate light yellow shades obtained with herbs on cotton fabrics. Figures 6, 7, 8, and 9 show colours obtained along with the significant representative colouring component structure illustrated. Inherently, each herb would contain several phytochemicals.

#### Herbal Colours acquired on cotton fabrics 

The results gained on biomordanting the cotton fabrics with oak bark, dandelion leaves, nettle leaves, and bilberry leaves and afterwards coloured with violet herbs are depicted in pictures from Figs. 6, 7, 8, and 9.

In summary, the moderate extraction produced the above results for deeper shades strong herbal concoctions are recommended.

#### Mode of chelation

**Fig. 10:**
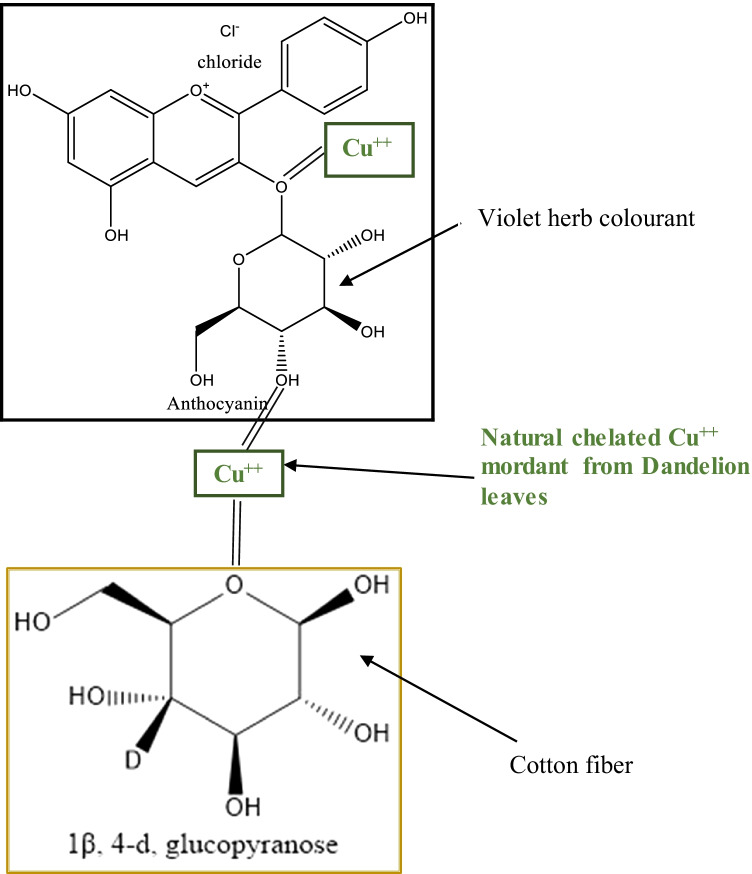
The plausible mode of chelation in-between the biomordant-fabric-colourant

The plausible mode of chelation as perceived for the current research is illustrated in Fig. 10 with an example of dandelion leaves as it was observed to be the highest in copper element content. The covalent bond formation occurs in-between the biomordant (dandelion leaves)-cotton fabric-colourant (violet herb).

The chemical structures are cited as gained from the PerkinElmer Sofware for the cotton fabric and the violet herb (Vukics et al. 2008; PerkinElmer Informatics 2017).

### Natural biomaterial analysis

The plant-based biomaterials were characterized on ATR-FTIR and FAAS. The results acquired are interpreted herein.

#### Fourier transform infrared spectroscopy (ATR-FTIR)

**Fig. 11:**
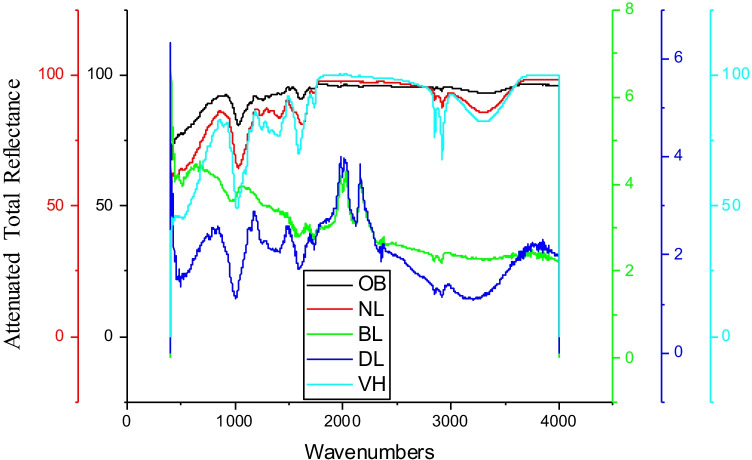
ATR-FTIR spectrums of various raw herbs

The raw herbs, biomordanted cotton fabric samples with herbs, and violet herb-coloured cotton fabric samples were analyzed using FTIR to identify functional groups imparted onto variously treated fabric samples as described herein. The ATR-FTIR analysis of raw herbs, namely nettle leaves, bilberry leaves, dandelion leaves, and the violet herb, is shown in Fig. 11, and the identification and interpretation of the functional groups are demonstrated in Table 6.

**Table 6:** ATR-FTIR examination of raw herbs in research

Herbal biomaterial	Wavenumbers (cm^−1)^	Strength of peak	Functional group
Nettle leaves, NH	520.07 to 593.59 cm^−1^	Moderate	C–Br stretch of alkyl halides
	1026.82 and 1047.34 cm^−1^	Moderate	C-N stretch of aliphatic amines
Bilberry leaves, BL	955.36 cm^−1^	Strong	= C-H bend of alkene
	1554.87 cm^−1^	Strong	N–O asymmetric stretch of nitro compounds
	1729.82 cm^−1^	Strong	C = O stretch of α and β unsaturated esters
	2117.78 cm^−1^	Weak	-C triple bond C – stretch of alkynes
	3367.22 cm^−1^	Moderate	C-H stretch of alkanes
Dandelion leaves, DL	767.75 cm^−−1^	Moderate	C–Cl stretch of alkyl halides
	1003.05 cm^−1^	Strong	carboxylic acids C-O stretch
	1414.73 cm^−1^ and 1592.30 cm^−1^	Moderate	C–C stretches of aromatics
	1730.75 cm^−1^	Strong	C = O stretch of aldehydes and aliphatic
	2116.96 cm^−1^	Weak	-C = C- stretch of alkynes
	2914.98 cm^−1^	Moderate	C-H stretch of alkanes
Violet herb, VH	520.48 and 553.25 cm^−1^	Moderate	C–Br stretch of alkyl halides
	1016.00 cm^−1^ and 1097.62 cm^−1^	Strong	carboxylic acids C-O stretch
	2917.21 cm^−1^	Moderate	C-H stretch of alkanes

**Fig. 12:**
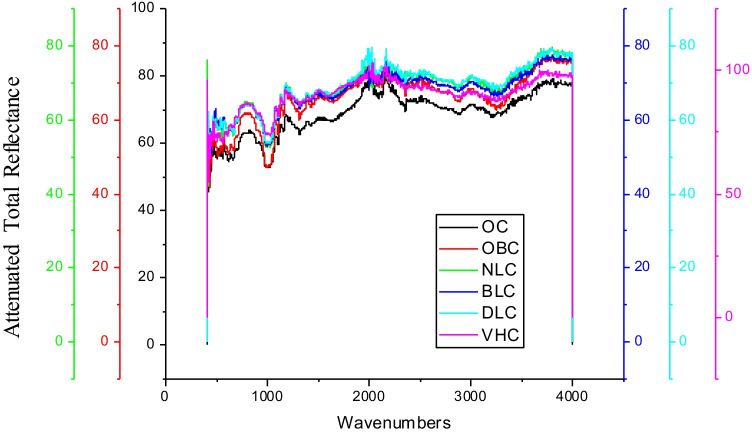
ATR-FTIR spectrums of biomordanted cotton fabric samples

The functional groups inherently present in herbs in research, namely, nettle leaves, bilberry leaves, and dandelion leaves, formulate covalent bonds with cotton fabric, functional groups, to further form a complex with violet herb colouring component thereby forming biomordant-cotton fabric-colourant complex. Hence, a large complex is formed within the microfibrillar structure. The functional and fastness properties of the resultant fabric are directly proportional to the number of large complexes formed within the microfibrillar structures of the cotton fabric. The ATR-FTIR of raw herbs indicated bilberry leaves and dandelion leaves to be the densest of all in varied functional groups. The ATR-FTIR analysis of cotton fabric processed with biomordants, namely nettle leaves, bilberry leaves, dandelion leaves, and the violet herb, is shown in Fig. 12, and the classification and analysis of the functional groups are exhibited in Table 7.

**Table 7:** ATR-FTIR investigation of biomordanted cotton fabrics in research

Herbal biomaterial	Wavenumbers (cm^−1^)	Strength of peak	Functional group
NLC	520.49 to 663.18 cm^−1^	Moderate	C–Br stretch of alkyl halides
	691.29 cm^−1^	Strong	alkynes C-H bend
	981.81 cm^−1^	Strong	alkene = C-H bend
	1000.52 to 1104.02 cm^−1^	Strong	carboxylic acids C-O stretch
BLC	517.33 to 664.52 cm^−1^	Moderate	C–Br stretch of alkyl halides
	981.85 cm^−1^ and 999.01 cm^−1^	Strong	alkenes = C-H bend
	1026.70 and 1049.94 cm^−1^	Moderate	aliphatic amines C-N stretch
DLC	515.23 to 663.66 cm^−1^	Moderate	C–Br stretch of alkyl halides
	897.15 to 997.70 cm^−1^	Strong	alkenes = C-H bend
	1024.89 to 1104.10 cm^−1^	Strong	C-N stretch of aliphatic amines
VHC	518.39 to 663.49 cm^−1^	Moderate	alkyl halides C–Br stretch
	982.49 to 995.93 cm^−1^	Strong	= C-H bend of alkenes
	1024.86 cm^−1^ and 1050.03 cm^−1^	Moderate	aliphatic amines C-N stretch

**Fig. 13:**
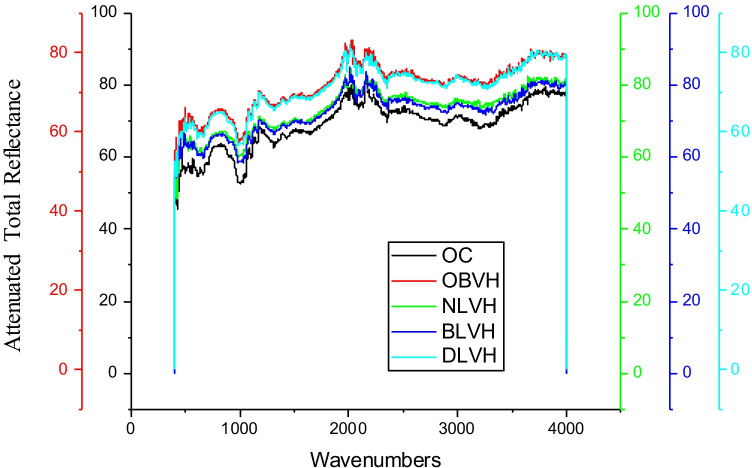
ATR-FTIR spectrums of Biomordanted and coloured cotton fabrics

The ATR-FTIR analysis of the cotton fabrics pre-mordanted with herbs such as nettle leaves, bilberry leaves, and dandelion leaves, and afterwards coloured with the violet herb is shown in Fig. 13, and the identification and interpretation of the functional groups are demonstrated in Table 8.

**Table 8:** ATR-FTIR investigation of biomordanted and coloured cotton fabrics in research

Herbal biomaterial	Wavenumbers (cm^−1^)	Strength of peak	Functional group
NLVH	520.54 to 690.96 cm^−1^	Moderate	C–Br stretch of alkyl halides
	712.92 cm^−1^ to 763.84 cm^−1^	Moderate	C–Cl stretch of alkyl halides
	899.99 cm^−1^	Strong	C-H “oop” of aromatics
	915.44 cm^−1^	Moderate	O–H bend of carboxylic acids
	982.55 cm^−1^	Strong	= C-H bend of alkenes
	1024.42 cm^−1^, 1050.24 cm^−1^, and 1101.80 cm^−1^	Moderate	C-N stretch of aliphatic amines
BLVH	514.12 to 663.60 cm^−1^	Moderate	C–Br single bonds of alkyl halides
	701.00 to 796.79 cm^−1^ and 816.10 to 897.80 cm^−1^	Strong	C-H “oop” bands aka out-of-plane bands of aromatics
	915.69, 996.50, and 982.50 cm^−1^	Strong	= C-H bend of alkenes
	1025.59, 1050.41, and 1103.54 cm^−1^	Moderate	C-N stretches of aliphatic amines
	1277.30, 1311.75, and 1334.10 cm^−1^	Strong	C-N stretch of the aromatic amines
DLVH	551.35 to 688.55 cm^−1^	Moderate	C–Br stretch of alkyl halides
	745.37 to 891.00 cm^−1^	Strong	= C-H bend of alkenes
	900.43, 916.48, and 981.72 cm^−1^	Strong	C-O stretch of alcohols, carboxylic acids, esters, and ethers

The functional groups manifested on cotton fabric samples pre-treated with herbs and subsequently coloured with violet herbs as shown in Fig. 13 were denser than those exhibited on the cotton fabric samples pre-treated with herbs (Not subsequently coloured) as shown in Fig. 12. The ATR-FTIR of cotton fabric samples pre-treated with bilberry leaves and dandelion leaves and sequencially coloured with violet herb was the densest of all in varied functional groups like as examined in receptive raw herbs. The developed herbal treatment process provided cotton fabric with specific functionality. Therefore, a clinical investigation on the same is recommended for the future.

#### Atomic absorption spectrometry (AAS) analysis

**Fig. 14:**
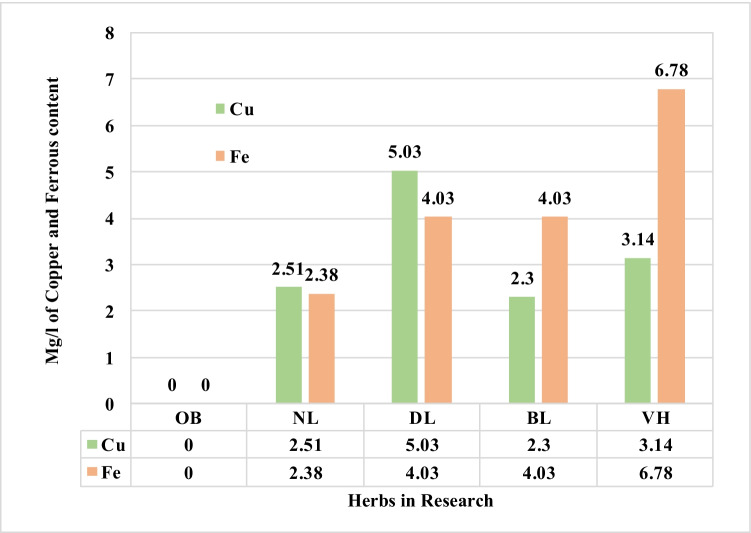
AAS analysis of natural biomaterials in research for copper and ferrous content

The AAS analysis of the herbs revealed the amount of copper and ferrous content on each of the herbs, namely dandelion leaves, nettle leaves, and bilberry leaves; refer to Fig. 14.

Interestingly it was the colourant, the violet herb, that exhibited the maximum ferrous content of 6.78 mg/l followed by bilberry and dandelion leaves each with 4.03 mg/l of ferrous content. Likewise, the dandelion leaves contained high copper content of 5.03 mg/l followed by violet herb and nettle leaves having a copper content of 3.14 and 2.51 mg/l, respectively. The copper and ferrous inherently present in herbs assist in enhanced fixation of plant-based colour on natural textile substrates as evaluated further. The copper and ferrous content in herbs deepen the shade obtained as noted in the K/S and b* colour values obtained with herbs in research as compared to the standard oak bark treated cotton fabrics.

### UV–vis spectroscopy analysis

**Fig. 15:**
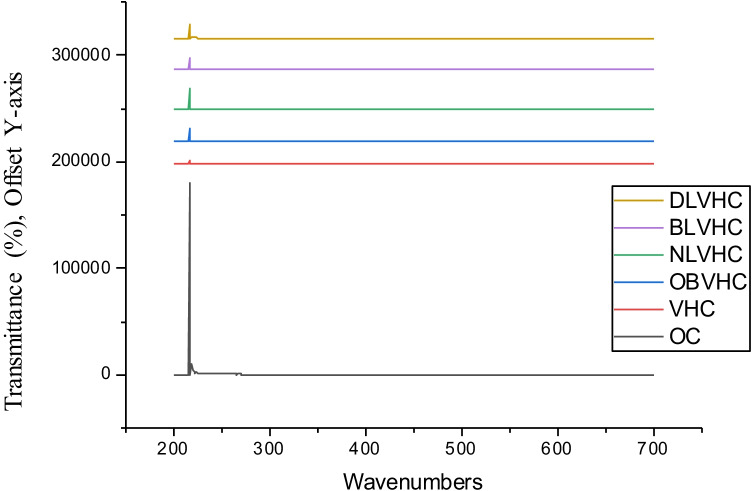
UV–Vis’s analysis of the herbs treated cotton fabric samples

The UV protection property of the herbs treated cotton fabric samples was assessed; refer to Fig. 15.

The ultraviolet ray’s transmittance percentage values obtained of the cotton fabrics processed with the mild herbal solution at varied wavenumbers are listed in Table 9. It could be noted that the lowest UVA transmittance value of 6.96% is obtained with the cotton fabric sample pre-treated with bilberry leaves and coloured with violet herbs. Likewise, the cotton fabric sample pre-treated with dandelion leaves and nettle leaves had 7.55% and 11.02% of UVA transmittance, respectively. A similar trend was identified with the % transmittance values for the UVB region. The minimum UVB transmittance percentage was noted to be 12.04% of the cotton fabric biomordanted with bilberry leaves and coloured with the violet herb. Thereafter, the second-minimum value of 14.88% of UVB transmittance was noted for the cotton fabric biomordanted with dandelion leaves and coloured with the violet herb. And lastly, the values of 21.72% of UVB transmittance was observed for the cotton fabric biomordanted with nettle leaves afterwards coloured with the violet herb. Ideally, the UVB transmittance percentage scale as given by the British Standards Institution (BSI) is illustrated in Table 10. The previous study by Sarkar evaluated UPF of cotton fabrics coloured with madder, indigo, and cochineal, and resultant fabrics were concluded to be excellent in UVP ability. Likewise, it was established that darker hues with a high concentration of natural colour on the fabric are more UV protective (Sarkar 2004).

**Table 9:** The UV-transmittance percentage values as obtained from herbs treated cotton fabric samples

Wavenumbers (nm)	Effects	Transmittance (%)					
		OC	VHC	OBVHC	NLVHC	BLVHC	DLVHC
VR—700–401	No harm	37.28	26.22	25.61	30.75	28.09	28.53
UVA—315–400	Damage skin	67.26	4.37	8.03	11.02	6.96	7.55
UVB—314–280	Damage skin	98.93	7.69	15.41	21.72	12.04	14.88
UVC—200–279	Most harmful	99.95	9.60	15.03	25.90	13.21	19.96

**Table 10:** UVB transmittance percentage scale by BSI (The British Standards Institution 2012; Louris et al. 2018)

Ultraviolet protection scale	UVB transmittance percentage (%)
Excellent	Less than 2.5
Very good	3.3–2.5
Good	5.0–3.4

The UV protection percentage (%) scale is shown in Table 11.

**Table 11:** UV protection percentage (%) scale (Bonet-Aracil et al. 2016; Sin-hee 2006)

UPF	UVR block percentage (%)	Performance
15–24	93–96%	Good
25–39	96–97%	Very good
40–50 +	97% +	Excellent

UPF is ultraviolet protection factor and UVR is ultraviolet region, including UVA and UVB in this.

Among the cotton fabrics treated with new sources of herbal biomaterials, referring to Table 12, the ultraviolet protection offered by BLVHC is indicated to be the maximum of 93.04% in the UVA region and 87.96% in the UVB region. Conclusively, the low UV-transmittance values make the fabrics nearly suitable for summer wear. High saturation decoctions would further lower the transmittance percentage. It is worth noting that UVC does not reach the earth’s surface as most of it is absorbed by the atmosphere.

**Table 12:** The UVA and UVB protection percentage of herbal fabrics

Herbal fabrics	UVA protection percentage (%)	UVB protection percentage (%)
OBVHC	91.97	84.59
NLVHC	88.98	78.28
BLVHC	93.04	87.96
DLVHC	92.45	85.12

### Ecological parameters evaluated

The potential of hydrogen (pH), dissolved oxygen (DO), and oxidation–reduction potential (ORP) levels were noted of the spent solution after processing the cotton fabrics with standard oak bark biomordant and nettle leaves, dandelion leaves, and bilberry leaves innovative biomordants in research (Table 13). Note, DW is the distilled water.

**Table 13:** The pH, DO, and ORP after mordanting with herbs

Plant extract	pH	Millivolts (mV)	Leftover solution DO (mg/l)
DW	6.90	54.3	6.90
OBC	4.33	100.0	7.91
NLC	8.30	− 82.9	3.73
DLC	5.51	72.5	6.54
BLC	4.28	141.2	7.30

DW is distilled water, C is cotton fabric, OB is oak bark, NL is nettle leaves, DL is dandelion leaves and BL is bilberry leaves

It is conspicuous that the spent solution after processing the cotton fabric samples with nettle leaves acquired a negative OPR value of − 82.9 mV and a low DO value of 3.73 mg/l hence unfit for disposal into water bodies. It is therefore suggested to treat the spent solution at the wastewater treatment plant before further upscaling. The DO level of 6.54 mg/l and 7.30 mg/l was gained with dandelion leaves and bilberry leaves spent solution that was in the acceptable array hence sustainable. The ORP values were 141.2 mV and 72.5 mV for bilberry leaves and dandelion leaves correspondingly hence capable to oxidize contaminants if any and therefore ecological. The sustainable spent solution could be reused or recycled or biodegraded safely.

The pH of the spent solution was alkaline at 8.30 with nettle leaves. The pH of the spent solution was acidic at 5.51 and 4.28 with dandelion leaves and bilberry leaves, respectively. It is therefore recommended to neutralize the spent solution with organic calcium carbonate or acetic acid before disposing of it.

The DO of the spent solution after the colouration of cotton fabrics with violet herbs remained in an acceptable range therefore suitable for upscaling; hence, the materials and methods in research are sustainable; refer to Table 8. Likewise, it is significant to note that the positive value of ORP of the spent solution increased as compared to the original distilled water ORP with the highest obtained of cotton fabrics pre-treated with bilberry leaves and subsequently coloured with violet herbs having an ORP of 101.3 mV (Table 14). Therefore, it can be concluded the herbs and methods of processing developed in this research are highly conducive to the environment and human health alike. The absurdity is that the textile industry starts the wet process input with a cheap and unreliable source of water that has no or extremely low dissolved oxygen levels. Therefore, we can estimate what happened to rivers in manufacturing countries, namely the Bandi River and Krishna River of India and Citarum River of Indonesia, and the plight of people and surrounding flora and fauna. It is high time to correct our ways and protect the natural resources as the rush for cheap has massively traded upon humanity and the environment.

**Table 14:** The pH, DO, and ORP of solutions after colouration with the violet herb

Plant extract	pH	ORP	DO
DW	6.36	32.3	7.75
VHC	5.09	98.3	9.68
OB + VHC	5.05	100.7	10.56
NL + VHC	5.19	96.0	7.96
DL + VHC	5.13	98.8	7.77
BL + VHC	5.08	101.3	7.90

### Evaluation of fastness properties

Analysis with Blue Wool Standard, fair lightfastness rating of 4 was obtained for all the treated cotton fabric samples. For greyscale analysis, the wash fastness to colour change was poor as rated 2 for all the cotton fabric samples processed with mild herbal infusions. The wash fastness to colour staining was very good with a rating of 4–5 for all the treated cotton fabric samples (Table 15). Likewise, the dry and wet rub fastness values obtained were 4–5 and 4, implying very good and good, respectively. Overall consistent results were obtained. The treatment with pure extract of copper and ferrous from the violet herb, dandelion leaves, and bilberry leaves would considerably assist in deepening the shades and acquiring good to excellent wash fastness to colour-change properties. Equally, if not pure extracts merely considering strong infusions of herbal solutions would significantly enhance the fastness properties.

**Table 15:** Fastness properties of cotton fabric samples mordanted and coloured with herbal materials

Samples	Wash fastness		Light	Rub-dry	Rub-wet
	Colour change	Colour staining			
VH	2	4–5	4	4–5	4
OB + VH	2–3	4–5	4	4–5	4
MH + HF	2	4–5	4	4–5	4
RH + HF	2	4–5	4	4–5	4
BCS + HF	2	4–5	4	4–5	4

### ANOVA one*-*way test for validation of hypothesis

**Fig. 16:**
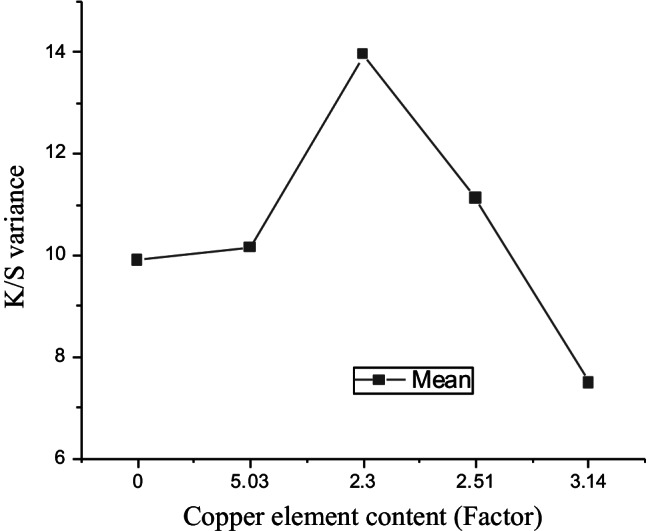
Means plot SE as an error for (factor) copper element and (response) K/S variance analysis

**Fig. 17:**
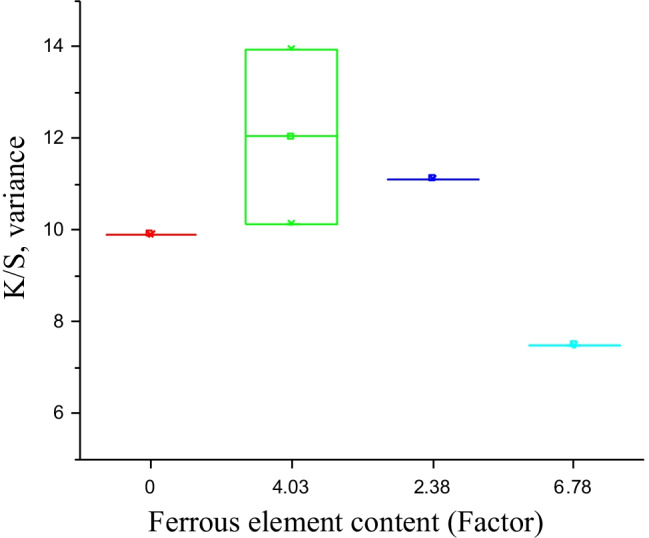
Box chart for (factor) ferrous element content and (response) K/S variance analysis

The ANOVA one-way test was performed to analyze the impact of copper and ferrous element content individually on K/S values obtained on cotton fabric samples treated with the corresponding herbs as tabled in Table 16. The representative graphs are illustrated in Figs. 16 and 17.

**Table 16:** Descriptive data for ANOVA one-way analysis

Herbs	Cu^++^	Fe^+++^	K/S
Sample name	Independent variable *x*-axis	Independent variable *x*-axis	Dependent variable *y*-axis
OB	0	0	9.90
DL	**5.03**	4.03	**10.14**
BL	2.3	4.03	13.95
NL	2.51	2.38	11.12
VH	3.14	**6.78**	7.49

Figure 16 demonstrates the mean standard error plot for copper element and K/S variance analysis gained on herbal fabrics biomordanted with oak bark, nettle leaves, bilberry leaves, and dandelion leaves and afterwards coloured with the violet herb. Triplicate readings were utilsed, the analysis validates that at 0.05 (95% of the confidence level), the population variance is significantly different and therefore no random error as noted in Fig. 16. The probability value of 0.003 and 0.005 were acquired for copper and ferrous element analysis (on K/S) respectively. Therefore, the results are statistically significant. Similarly, the actual power indicated that the experiential results are 95% trustworthy.

The box chart in Fig. 17 indicates the impact of ferrous element content on the K/S variance analysis.

Overall, the ANOVA one-way analysis reinforce that DL + VH processed cotton fabric samples to be remarkably noteworthy among all herbs. The DL is high in copper element content and its pair with VH is high in ferrous element content that delivers the processed cotton fabric with a high K/S value of 10.14 as denoted with values in bold in Table 16. Therefore, the combination of DL + VH makes a good pair. The mode of chelation depicted in Fig. 10 represents the respective complex formations, namely C-DL-VH. It is essential to note that the K/S values of 13.95 and 11.12 gained on cotton fabric samples processed with bilberry and nettle leaves correspondingly could be attributed to the presence of other phytochemicals namely kaempferol in bilberry leaves and coproporphyrin in nettle leaves as illustrated in Figs. 7 and 8, respectively. The copper and ferrous element contents in bilberry and nettle leaves are relatively low as demonstrated in Fig. 14 and Table 16 Even, the BLVH and NLVH treated fabrics are prospective herbal cotton fabrics.

## Conclusions

To navigate our ways through the troubled water, earth, and air due to emissions and effluents from the textile industry, the research derives noteworthy sustainable outcomes summarized as follows:

1. The method of extraction, biomordanting, and colouration with new sources of herbs, namely dandelion leaves, bilberry leaves, nettle leaves, and violet herb, was ecologically applied on cotton fabrics with sustainable outcomes. The method devised was low on water and energy demands hence sustainable. The processing at 60°C and below would protect the original colour and functional phytochemicals intrinsically enclosed in each herb, which is otherwise expended at a temperature above 60°C.
2. The maximum K/S values of 13.95 were gained with cotton fabric pre-treated with bilberry leaves and subsequently coloured with violet herbs; likewise, the highest b* value of 19.12 was noted with the same.
3. The ATR-FTIR peak analysis noted strong = C-H bend of alkenes, strong C = O stretch of α and β unsaturated esters, and strong C-O stretch of carboxylic acids functional groups in raw herbs and its treated cotton fabrics. The cotton fabrics pre-treated with bilberry leaves and dandelion leaves and sequentially coloured with violet herbs were the densest in the functional groups, therefore of potential therapeutic benefits to the wearer.
4. The AAS analysis indicated the substantial presence of ferrous content of 6.78 mg/l and copper content of 5.03 mg/l in violet herbs and dandelion leaves, respectively, hence suitable for prospective applications as biomordants and colourants for textile substrates. As an implication, it would prevent the depletion of mineral ores hence a sustainable alternative to fossil-based copper and ferrous elements.
5. It could be noted that the lowest UVA % transmittance value of 6.96% and UVB % transmittance value of 12.04% was obtained with the cotton fabrics pre-treated with bilberry leaves and coloured with violet herbs. In other words, the ultraviolet % protection was 93.04% and 87.96% in the UVA and UVB of UV regions, respectively, for the cotton fabrics biomordanted with bilberry leaves and coloured with the violet herb, hence suitable for summer wear.
6. It is conspicuous that the spent solution after processing the cotton fabrics with nettle leaves acquired a negative OPR value of − 82.9 mV, a low DO value of 3.73 mg/l, and alkaline pH of 8.30, hence unfit for disposal into water bodies. Interestingly the spent solution after colouration with violet herb of nettle herb pre-treated cotton fabric had the DO of 7.96 mg/l and positive ORP of 96.0 mV and pH 5.19, hence sustainable for further disposal or recycling or upscaling.
7. A fair lightfastness rating was obtained on the blue wool scale for all the cotton fabrics pre-treated and coloured with herbs in research.
8. Very good and good dry and wet rub fastness properties were respectively obtained for all the cotton fabrics pre-treated and coloured with herbs in research.
9. Likewise, the wash fastness to colour change was poor, and wash fastness to colour staining was very good for all the cotton fabrics pre-treated and coloured with herbs in research. Therefore, it is recommended to utilize strong decoctions. The surface modification techniques such as plasma surface modification would plausibly propel fastness properties and functional properties alike.
10. The ANOVA one-way test concluded the absorbance (K/S) value of the cotton fabric processed with dandelion leaves and afterwards coloured with the violet herb to the significantly shaped by the copper and ferrous element contents inherently present in the corresponding herbs.

**Fig. 18:**
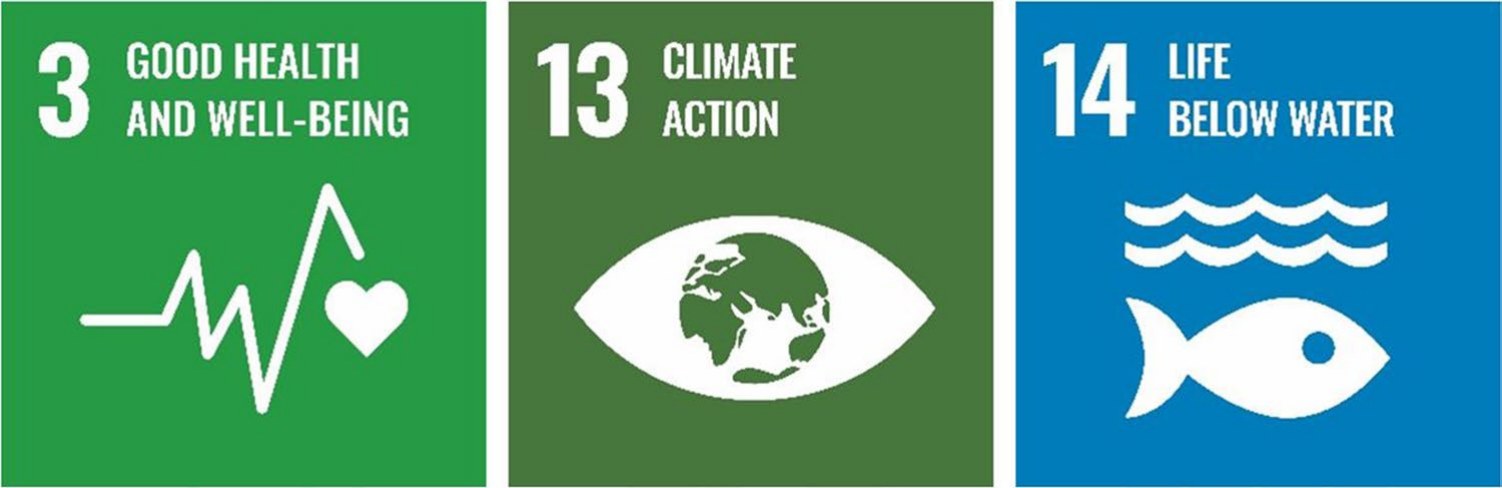
Sustainable development goals are addressed in research (United Nations n.d.)

Overall, like a drop in an ocean though the ecological research outcomes propel sustainability in textiles adhering to Sustainable Development Goals (SDG’s) as shown in Fig. 18.

## Future work

**Fig. 19:**
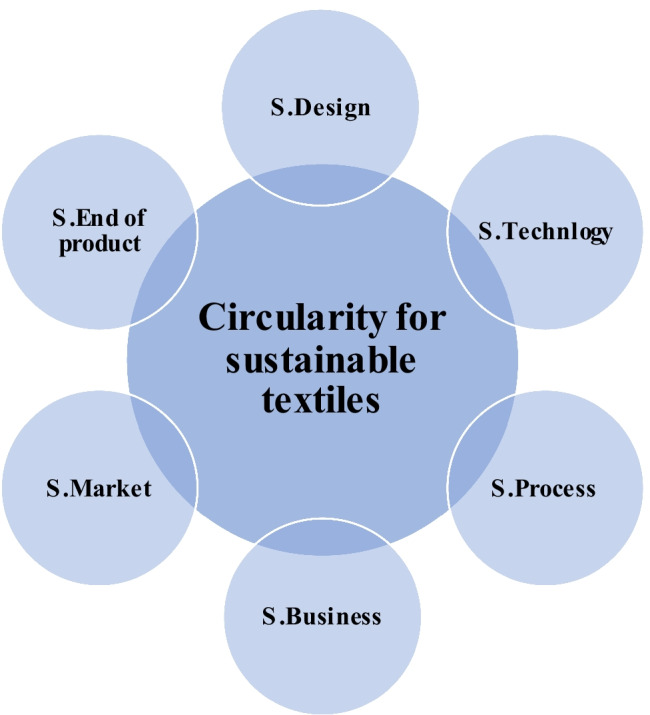
Sustainable circularity is predicted for the sustainable textile industry (UN Sustainable Development Goals 2020)

The circularity of the textile industry in the true sense is changing the DNA of textile manufacturing that necessitates internalizing the best sustainability practices. A holistic framework integrates imperative aspects as depicted in Fig. 19, namely sustainable (S) design, sustainable processing technology, sustainable business supply chain, sustainable market consumption, and sustainable zero-carbon deposal of the product. A successful circularity imperatively requires keeping clean processing technology at its heart, hence the instigated research that impels sustainable circularity (UN Sustainable Development Goals 2020).

To sum up, realistically we need to realize that there is no “Planet B”, and hope lies in living life in harmony with the laws of nature (Glover 2020). The study utilized mild infusions for pale shades. However, for strong and optimum infusions, 4 h plus infusion time is also recommended in the literature. Increasing the processing time and depth of shade would further enhance the colourimetric values and fastness properties. Also, pure extracts of plant-based copper and ferrous element inherently existing in the chelated form would impel colour values, fastness properties, and functional properties by manifolds, hence suggested for future research and development. Likewise, life cycle assessment and clinical investigation of potential functional benefits of herbal fabrics to the wearers are recommended for the future.

## Data Availability

The authors have duly acknowledged data and materials in research.
